# Energy Storage as an Equity Asset

**DOI:** 10.1007/s40518-021-00184-6

**Published:** 2021-05-20

**Authors:** Bethel Tarekegne, Rebecca O’Neil, Jeremy Twitchell

**Affiliations:** Pacific Northwest National Laboratory, Richland, WA USA

**Keywords:** Energy storage, Equity, Energy justice, Clean energy transitions, Energy policy

## Abstract

**Purpose of Review:**

This review offers a discussion on how energy storage deployment advances equitable outcomes for the power system. It catalogues the four tenets of the energy justice concept—distributive, recognition, procedural, and restorative—and shows how they relate to inequities in energy affordability, availability, due process, sustainability, and responsibility.

**Recent Findings:**

Energy storage systems have been deployed to support grid reliability and renewable resource integration, but there is additional emerging value in considering the connections between energy storage applications and equity challenges in the power system. Through a thorough review of the energy justice and energy transitions literature, this paper offers the equity dimensions of storage project design and implementations.

**Summary:**

Emerging energy programs and projects are utilizing energy storage in pursuit of improved equity outcomes. Future research and policy design should integrate energy justice principles to align storage penetration with desired equity outcomes.

## Introduction

Public interest and regulatory efforts that target the climate crisis and advance an energy transition that leaves no one behind have started to increase at all levels. For example, at the federal level, the House Select Committee on the Climate Crisis offered a climate crisis action plan that sets a goal of an economy-wide net zero emissions by 2050 and the reduction of pollution in environmental justice (EJ) communities [1]. State-level climate action is also accelerating with 15 states and territories aiming to move towards a 100% clean energy future [2]. For example, New York state’s economy-wide climate law requires 70% renewable energy (RE) in the electricity sector by 2030 with 35% of clean energy revenue flowing to underserved communities and a broader goal of 100% carbon-free electricity by 2040 [3••]. Similarly, New Jersey’s Board of Public Utilities (NJBPU) recently developed an Office of Clean Energy Equity that is tasked with overseeing the distribution of clean energy technologies to ensure equitable access by all residents [4].

A key enabler in the future of this decarbonized and renewable energy (RE)–dominated power system is the integration of energy storage. Energy storage technologies—pumped hydropower, battery storage, flywheel—mitigate the non-dispatchable production of RE by storing the energy output for use when needed. Recently, large-scale battery storage has seen an increasing penetration in the power grid [5]. Energy storage systems (ESS) can be integrated at various points on the grid. ESS can be located at the transmission level to relieve congestion, at the distribution level to improve reliability, and behind-the-meter (BTM) to relieve targeted congestion and provide load reduction. The flexibility in storage deployment at the point of demand or at the grid scale provides convenience and quick response in matching supply and demand. This enhanced system operation lowers peak demand and leads to a reduction in the energy burden on consumers [3••]. In cases where extreme weather events could affect the reliability of the power infrastructure, storage can maintain electric service, support critical loads, and enhance grid resilience.

A valuable, but less examined, benefit of energy storage is its ability to contribute to the just energy transition. The concept of just energy transition alludes to a process of adding justice and equity concerns in the energy transition from high-carbon energy sources to a renewable energy–dominated resource portfolio [6]. Specifically, a just energy transition focuses on striving to ensure that the costs and benefits of the transition are equitably shared [7]. Absent that focus, the clean energy transition could perpetuate the inequities of the pre-existing centralized and fossil fuel–based power system: between those consumers, businesses, and industries that can take advantage of cleaner sources of energy, emissions reductions, and the employment opportunities of the transition; and those that will disproportionally bear the burden of the transition and lack access to the opportunities of the transition. Centralized and distributed energy systems have varying levels of capabilities in advancing an equitable energy transition. Historically, the reliance on centralized systems has resulted in inequitable outcomes in relation to resource depletion, GHG emissions, power system unreliability, and limited access to energy production and consumption by underserved communities [8], whereas distributed systems exhibit a capability to enable equitable outcomes in relation to local energy production (prosumers), community self-sufficiency, grid flexibility, reliability, and resilience [8].

## Methodology of Review

This paper assesses the equity-storage linkage to identify the equity implications of further integrating storage in the power system. The next section starts by providing a discussion of the existing power system inequities. Then, the paper offers the four tenets of energy justice—distributive, recognition, procedural, and restorative—to examine the role of energy storage in advancing a just energy transition. Within the broader four justice tenets, the paper relies on the affordability, availability, due process, sustainability, and responsibility energy justice principles to help identify the concerns or inequities of the energy system. In this paper, the tenets and principles are linked as follows: distributive justice is assessed through the affordability and availability principles; recognition and procedural justices are assessed through the due process principle; and restorative justice is assessed through the sustainability and responsibility principles. In doing so, the paper examines the temporal, economic, socio-political, geographic, and technological equity dimensions of the power system and shows where and how storage fits in the discussion. This review paper concludes with a recommendation of future research and practice areas to embed storage as an equity enabling technology.

## Equity in the Power System

The inequities of the power system are usually manifested in two ways: the lack of access to the energy transition technology opportunities and the inequitable distribution of benefits [9–11]. The first inequity relates to the expensive capital cost of the new technologies [12, 13] and the underlying affordability implications in the cost of energy when utility rates shift to lower-income ratepayers [14]. If these technologies—DER, transmission development, RE, digitization—are not available equitably to everyone, the inequities of these burdens will be disproportionate on those that cannot afford to transition. The benefits of the transition—low emissions, electric vehicles, photovoltaics (PV), community solar, smart meters, energy-efficient (EE) homes, and appliances—are mostly available for higher income households and are not universal. For instance, a household that can access and afford to install a rooftop PV array will benefit by reducing energy bills and shifting the costs of integration and investment, however small, to all customers uniformly, whereas the household that cannot afford to do so will not gain the benefit, but also is more likely to be needful of the bill reduction and face energy insecurity in the long term [15••, 16]. Energy insecurity, or the inability to meet basic household energy needs, may lead vulnerable households to forgo essential needs such as food and medical care [16]. The distribution of technology siting is also a concern where majority of the clean energy technologies are located on predominantly low-income and vulnerable community geographies [17, 18].

On the systemic and institutional level, the power system inequities relate to the opportunity to participate in decision-making processes [19, 20]. The energy transition decision-making procedures often do not include communities that host the infrastructures [18, 21]. Project acceptance and perception of fairness is often tied to the transparency and inclusivity of the decision process. An example here is the procedural inequity in the lack of advocacy for equitable distribution of welfare in utility commissions [15••]. Comprehensive targets for equity are crucial in decision-making processes to advance programs that address the underlying inequities in the system.

## Equity and Energy Storage

Energy equity refers to the distribution of costs and benefits of the energy power system. The energy literature engages with the issue of equity through the concept of energy justice [20]. The latter concept itself is highly contested and could have diverse meanings for various stakeholders [22]. In the broader sense, energy justice seeks to integrate justice principles, fairness, and social equity into energy systems [23]. It does so by evaluating where injustices occur, who gets to bear the injustices, what process are there to remedy the injustices, and how the injustices can be eliminated [10, 20].

The current energy justice scholarship summarizes these principles under the four core tenets of energy justice: distributive justice, recognition justice, procedural justice, and restorative justice [10, 19, 20, 24, 25]. Distributive justice involves asking the question—where are the injustices? In general, this is the practice of embedding justice in the siting of energy infrastructure and the distribution of economic benefits and burdens [20]. Studies of energy insecurity and poverty highlight the distributional burden of rising energy prices [26•]. These injustices are analytically examined through the affordability and availability principles, which will be discussed further in the next section. The recognition justice tenet focuses on considering who in society is ignored or misrepresented in the energy system [20]. It helps expose the unfair proximity of vulnerable groups—ethnic minorities or indigenous peoples, to power plants and the unfair share of energy insecurity among the disabled and aging population [27]. The third tenet, procedural justice, is about equity as public participation through the notions of transparency, accountability, and due process [19]. Just outcomes are envisioned here by encouraging local knowledge mobilization, community engagement, and increased informational disclosure [28]. The last tenet, restorative justice, which is the least studied in the literature, is about the response to those impacted by the inequities of energy systems by advancing the sustainability and responsibility principles [25]. The sustainability and responsibility principles are based on the notion that energy resources should not be depleted too quickly and that all nations ought to protect the social and natural environment and minimize energy-related threats [19]. Advancing these two principles, restorative justice ensures an equitable repair to the harm done on people and the environment.

For these reasons, the clean energy transition may deepen certain existing inequitable outcomes on different socio-economic groups, racial and ethnic minorities, genders, geographics, and other demographics [9, 15••, 29]. Energy storage could address the aforementioned justice tenets and support a more equitable low-carbon energy transition [3••].

### Tenet 1 Distributive Justice

Availability and affordability of energy systems and services are key to realizing distributive equity [15••]. Availability as an equity principle affirms that people should have sufficient, reliable, and quality access to energy systems. Similarly, the affordability principle affirms that low-income and other vulnerable communities need financial as well as physical access to make use of available energy systems.

In the USA, low-income households spend a high percentage of their income (approximately three times higher) on energy cost compared to their mid-high income household counterparts [26•]. This is further exacerbated among those that face an added systemic inequity due to race, gender, disability, and age [27, 30]. For example, people with disabilities might have an increased energy cost in the summer because they need their homes to be cooler throughout the day [27]. Similarly, the elderly population requires a narrower temperature range to maintain their health, increasing their energy vulnerability [27]. Energy insecurity or bearing the energy burden could often lead households into critical stress, fear, and mental health issues. According to the EIA, in 2015, 17 million households faced an energy disconnection notice [31]. Due to the COVID-19 pandemic, households have faced substantial energy burdens that can increase their vulnerabilities to the virus, psychological fatigue, and the potential loss of electricity due to non-payment as utility shutoff moratoriums come to an end [14, 32].

Another way to look at equity is by examining the accessibility of energy technologies across the socio-economic spectrum [15]. Tax credit–based incentives are not as favorable for communities that have low or no tax liability [33]. Financial structures that require high upfront costs or tolerance for extended payback periods, such as production incentives, will favor wealthier communities or businesses [13]. Renters, who do not control rooftops, and multifamily environments where electricity costs are typically not paid by property owners, create split incentive challenges for efficiency and rooftop solar installations. In contrast, rooftop solar has been available for over a decade for those who can afford the system and those with owner-occupied single-family housing [14].

As energy storage solutions become downscaled modular and available to energy consumers on a behind-the-meter basis [34], it may follow the same path as distributed solar installations and offer higher reliability and lower energy cost to individuals with disposable income. Storage installations could however be scaled and sited intentionally to address these inequities. Energy storage solutions could provide the means to reduce energy burden by curbing expensive demand charges, supporting community-serving facilities, and lowering energy costs to affordable housing residents [3••]. Localized storage deployments, or even an increased penetration in storage capacity, could replace fossil-fueled peaker plants that are often sited close to vulnerable communities [3••, 35]. An example is PG&E’s Oakland Clean Energy Initiative (OCEI) plan to replace an old fossil fuel power plant with clean energy solutions, energy storage, and electric system upgrades to empower local communities [36].

In relation to availability, storage supports grid reliability and resilience in response to natural disasters and other disruptions that require backup power [37]. To expand the distributive equity benefits of storage, incentives for storage deployment could target households that cannot access low-carbon energy systems and mitigate the inequitable distribution of clean energy technologies and benefits. For example, the California Public Utilities Commission (CPUC) runs a Self-Generation Incentive Program (SGIP) that offers energy storage rebates for homes, apartments, and critical facilities [38]. The CPUC authorized a $1 billion funding through 2024 for the SGIP with part of the prioritization being towards low-income and medically vulnerable customers. In addition, the SGIP provides higher rebates under the categories of “Equity” and “Equity Resiliency” lowering the cost of energy storage to almost free of cost to low-income households.

### Tenet 2 Recognition Justice and Tenet 3 Procedural Justice

For both recognition and procedural justice tenets, the assessment is on the processes of public participation: due process, representation, citizen engagement, and community consultation [20].

Recognition equity states that individuals must be fairly represented, and it reflects upon who exactly to focus on when thinking of energy victims [9, 20]. This tenet is critical for energy equity as it provides for the designation of specific solutions to address injustices. It asks the question, who is ignored? The answer identifies the sections of society that will be impacted or left behind in the transition. Minorities and indigenous people bear a disproportionate burden in energy production and power plant siting processes [9]. Indigenous peoples especially bear the misrecognition burden due to their reliance on land and resources that get impacted by extractive industries [39]. This inequity highlights the role of non-representation or non-inclusion of communities in the decision-making process, which points to the procedural equity tenet.

Procedural equity deals with the fairness of the decision-making process. Energy decision-making processes are highly complex. While there is a strong principle of third-party participation in the energy system, as captured for example in the Public Utility Regulatory Policies Act (PURPA) and the Open Access Transmission Tariff (OATT), or in independent market environments, participation still requires significant legal, financial, and engineering sophistication. To achieve procedural equity, decision-making requires full participation, adequate information, complete expression of opinion, and the impartiality of decision-makers.

The business model of aggregation of distributed energy resources is based on the integration of renewables onto the grid, in part spurred by storage systems’ potential to serve larger grid functions [40]. Energy system planning, on the other hand, is either held by utilities with obligations to serve and availability of rate-based investments with least-cost principles [41] or by entities concerned with grid-scale reliability. Siting practices are highly fractured based on fuel type and scale. For example, the Federal Energy Regulatory Commission (FERC) permits and oversees hydroelectric projects, Nuclear Regulatory Commission (NRC) permits nuclear projects, but states or counties will permit natural gas plants, wind, and solar projects depending on the size. Siting processes are initiated by independent operators and developers in response to financial and physical/fuel conditions, and so siting processes are standards driven and reactive.

Energy storage planning and siting could be executed in a way that advances recognition and procedural energy equity. Continued progress in battery storage research and development has increased the efficiency and safety of storage systems making them fairly attractive [42]. Through an inclusive energy decision-making process, states would be able to identify underserved and affected communities and design energy storage deployment mandates or consumer-based incentives to install storage to benefit those communities [3••]. This flips the idea that siting disproportionately harms indigenous populations, low-income communities, rural households, to one where local installation improves energy reliability and lowers costs.

As distributed energy resources (DER) transform the energy independence of communities, storage is a key enabler in the business model. Smaller grids with renewable resource-driven technologies are subject to more volatility without the ability to mitigate across a larger grid. Typically, this is managed with a dispatchable scalable resource such as a diesel generator. Storage is essential to maintaining non-emitting small grids, protection from fuel price spikes and supply chain shortages, while supporting independent renewable resource-driven systems [43]. These attributes can provide health, wealth, and financial protections that benefit vulnerable communities.

Storage business models that advance community wealth also have implications for recognition and procedural equity, consider implementation of community energy storage systems (CES) [44]. CES is an energy storage system designed with a community ownership and governance approach to generate socio-economic benefits. It is perceived as an intermediate solution between behind-the-meter storage systems and utility-scale distributed energy storage. The benefits of CES could be through an increased penetration of RE, the reduction in emissions, reduced energy bills, and the opportunity to generate revenue. Energy equity could be enhanced by strategically supporting the siting of CES in communities recognized as being impacted or regarded as underserved by the energy system. This could be done through co-ownership of storage assets with utilities or by subsidizing loans to low-income households to partake in CES.

Currently, there are two pathways for CES development—local and virtual [45]. A local CES exists in a specific physical territory within a local community. The members of the community could engage in CES development for energy independence, resilience, and energy security and they can own and self-govern the system. In the virtual CES business model, the participating communities would not need to be a specific local community. Participants in this pathway would be members who form virtual communities and use the CES to provide energy services on an aggregated basis. To advance the equity benefits of storage, States could enact incentive programs that aim to prioritize the preference of affected communities as sites for wealth-creating technologies, such as CES. For example, the California Energy Storage Initiative (SB 700) is designed to integrate revenue-generating systems in low-income and underserved communities [46]. The proposed SB 700 requires utilities to use 25% of the energy storage systems (ESS) money they receive in low-income neighborhoods and housing.

### Tenet 4 Restorative Justice

Advancing restorative equity aims to repair the harm inflicted by energy systems [15••, 25]. All power plants have an economic and environmental footprint. Fossil fuel extraction and production industries cause pollution with air quality and water quality effects. These same attributes have health effects on local communities [17, 39]. Renewable energy resources have also raised questions about interactions in the siting process, around land use, avian species, and flicker effects, as well as supply chain sustainability [15••].

Applying restorative equity works to reverse and repair the harms done in such scenarios. Storage is facilitating the clean energy transition by allowing more renewable energy to come onto the grid while maintaining reliable operation [42]. While it is not clear how much and what kind of storage is required for this trend to continue, storage will play a role in the continued grid-scale transition and consequent reductions in air emissions. In certain instances, storage can speed the total or incremental decommissioning of fossil fuel plants by allowing direct on-site replacement of power production in combination with other generators [3••]. However, it is crucial to proactively find solution to the potential inequities of storage in relation to scarce materials, working conditions for miners, and the disposal of batteries [47].

## Conclusion

In the context of energy systems, equity has two dimensions. The first relates to the lack of access to energy technologies. Low-income people and other minorities have been left out from accessing these resources due to systemic socio-economic barriers. The second is the inequitable distribution of the benefits and burdens energy systems create. These equity dimensions highlight the need to integrate justice concepts in the context of energy systems. This paper offered an equity framework to assess energy systems through the lens of the energy justice concept. It made the connection between energy system inequities and the capabilities offered by energy storage to enhance equitable outcomes.

In the changing energy landscape, energy storage is playing a critical role in maintaining the electric grid stability and reliability, and in expanding electric vehicles (EV) market penetration. The advancement in energy storage and the growth in the EV market will shape the future of the electric grid infrastructure, the transportation sector, and the broader regulatory and policy agenda in creating equitable synergies. Beyond the techno-economic capabilities of energy storage in grid system resilience, it can provide effective means to enhance equity in the power system. These benefits could be through improving public health, providing backup power during outages, decreasing the cost of energy and saving money for customers, and reducing environmental impact. To enhance the equity potential of energy storage, further research is needed in the techno-economic, and regulatory and policy dimensions. Research design should integrate energy justice principles to align storage penetration with desired equity outcomes.
